# Supplementary material for: Serum Levels of Dihomo-Gamma (γ)-Linolenic Acid (DGLA) Are Inversely Associated with Linoleic Acid and Total Death in Elderly Patients with a Recent Myocardial Infarction
Source: Nutrients. 2021 Sep 30;13(10):3475. doi: 10.3390/nu13103475 (PMC8540726; doi:10.3390/nu13103475)
Supplement: Supplementary file 1 [file nutrients-13-03475-s001.zip › nutrients-1367989-supplementary.pdf]

Supplementary Figure S1

CONSORT 2010 Flow Diagram

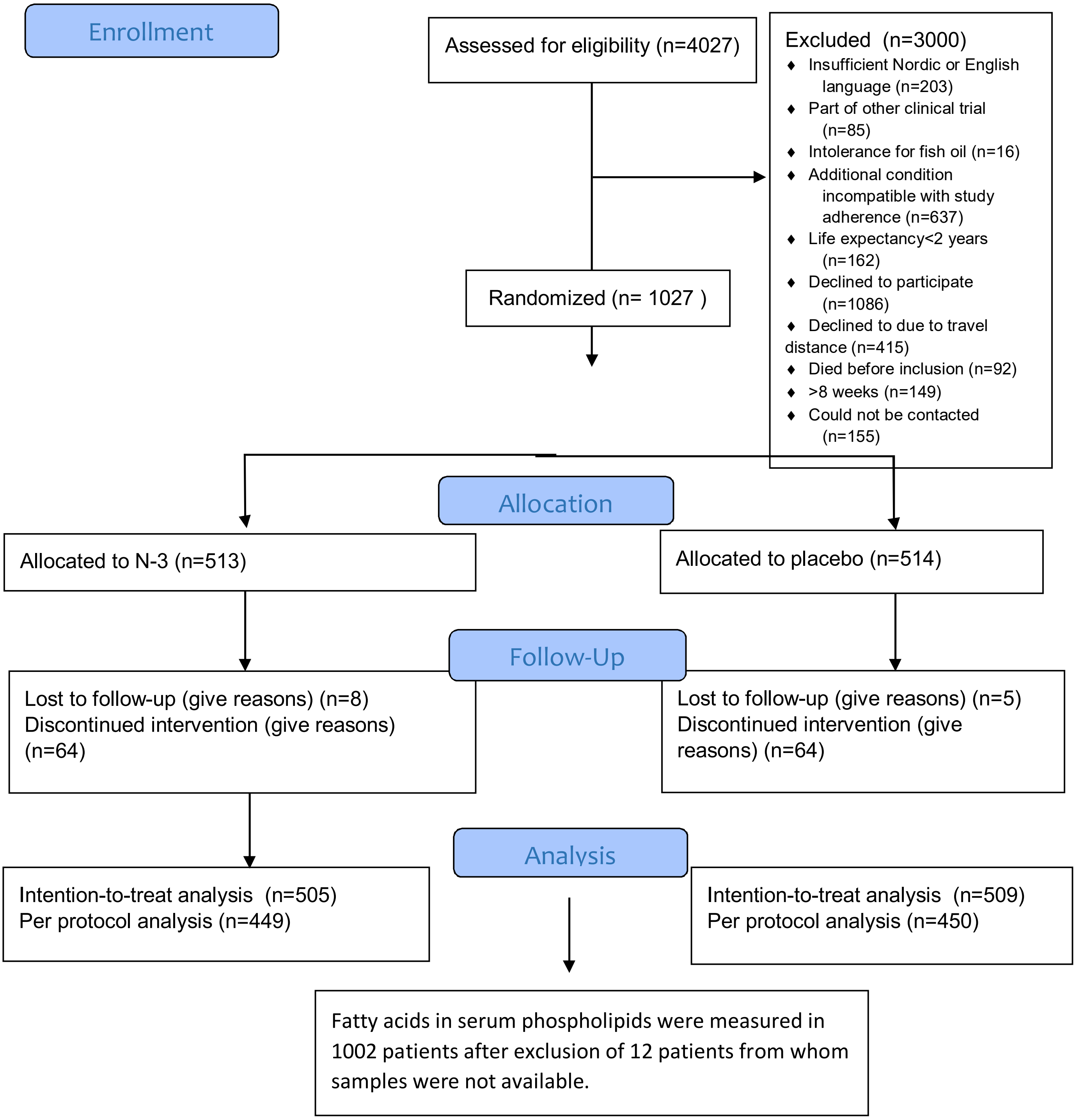

Supplementary Figure S2

Histogram showing the baseline distribution of DGLA measured in serum phospholipids, reported as percent of total fatty acids in 1002 patients in OMEMI.

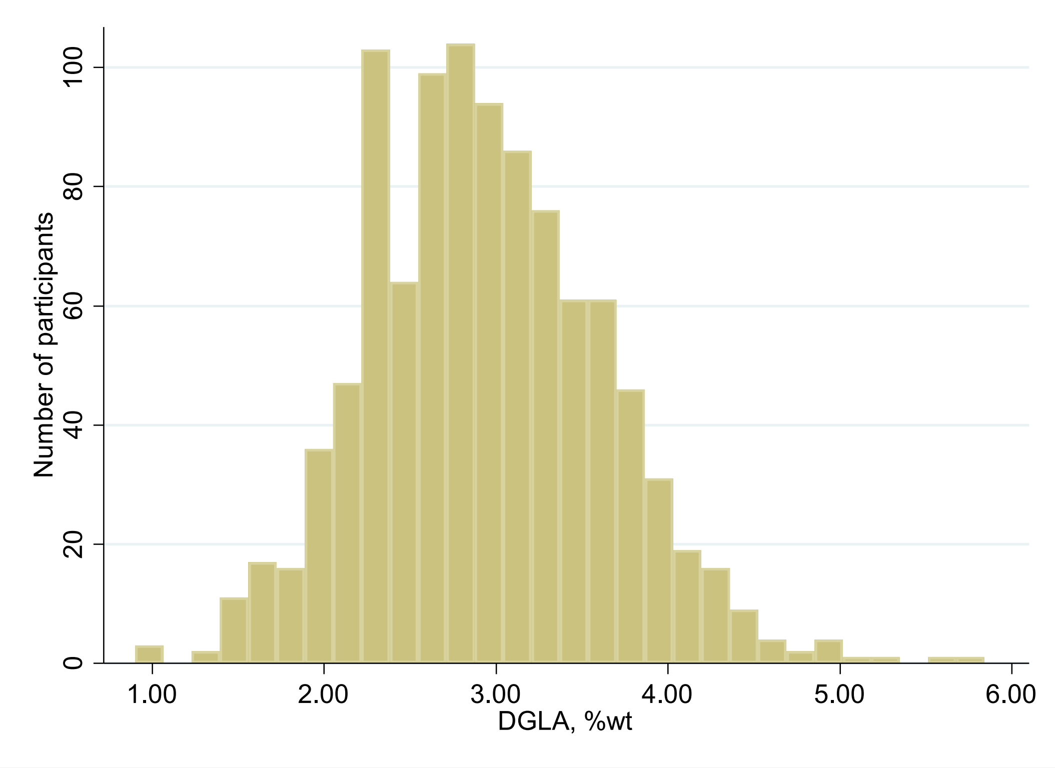

Supplementary Figure S3

Restricted cubic spline showing the relationship between DGLA (x-axis) and all-cause mortality (y-axis). P for non-linearity = 0.59

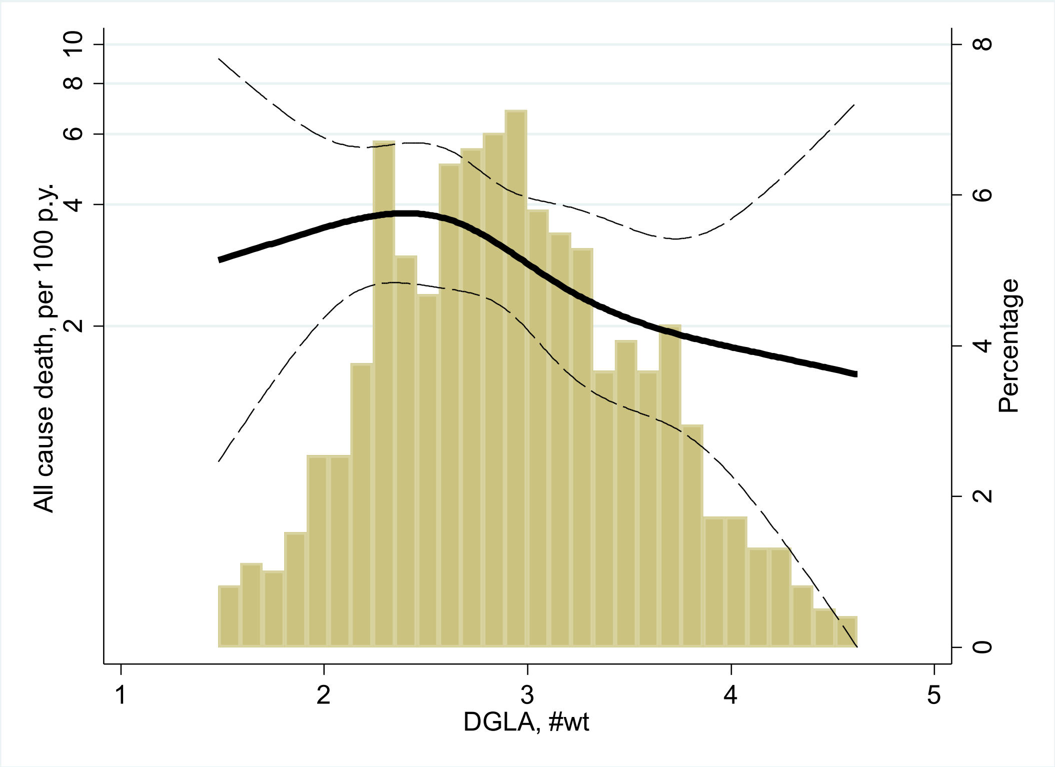

Supplementary Table S1. Endpoints by quartiles of linoleic acid (LA).

Total patient no: n=1002	**LA Q1** n=251		**LA Q2** n=251		**LA Q3** n=250		**LA Q4** n=250		**P-value for trend**
MACE	46	(18.3%)	54	(21.5%)	54	(21.6%)	54	(21.4%)	0.39
AMI	21	(8.4 %)	18	(7.2 %)	21	(8.4 %)	18	(7.2 %)	0.76
Revasc	17	(6.8%)	22	(8.8%)	18	(7.2%)	20	(8.0%)	0.78
Stroke	6	(2.4 %)	7	(2.8 %)	10	(4.0 %)	9	(3.6 %)	0.33
HF	12	(4.8 %)	11	(4.4 %)	9	(3.6 %)	13	(5.2 %)	0.94
Death	13	(5.2 %)	14	(5.6 %)	14	(5.6 %)	14	(5.6 %)	0.84

LA = Linoleic Acid. MACE = Major Adverse Clinical Events (nonfatal MI, unscheduled coronary revascularization, stroke, all-cause death, or hospitalization for new or worsened heart failure). AMI = Acute Myocardial Infarction. Revasc = Revascularization (percutaneous coronary intervention or coronary artery bypass grafting). HF = Heart Failure. P-values for trends across the quartiles.

Supplementary Table S2. Endpoints by quartiles of gamma linolenic acid (GLA)

Total patient no: n=1002	**GLA Q1** n=280		**GLA Q2** n=258		**GLA Q3** n=264		**GLA Q4** n=200		**P-value for trend**
MACE	59	(21.1%)	58	(22.5%)	49	(18.6%)	42	(21.0%)	0.68
AMI	17	(6.1 %)	22	(8.5 %)	19	(7.2 %)	20	(10.0 %)	0.19
Revasc	19	(6.8%)	23	(8.9%)	18	(6.8%)	17	(8.5%)	0.71
Stroke	15	(5.4 %)	10	(3.9 %)	4	(1.5 %)	3	(1.5 %)	0.005
HF	11	(3.9 %)	10	(3.9 %)	8	(3.0 %)	16	(8.0 %)	0.10
Death	17	(6.1 %)	14	(5.4 %)	15	(5.7 %)	9	(4.5 %)	0.52

LA=Gamma Linolenic Acid. MACE = Major Adverse Clinical Events (nonfatal MI, unscheduled coronary revascularization, stroke, all-cause death, or hospitalization for new or worsened heart failure). AMI = Acute Myocardial Infarction. Revasc = Revascularization (percutaneous coronary intervention or coronary artery bypass grafting). HF = Heart Failure. P-values for trends across the quartiles.

Supplementary Table S3. Endpoints by quartiles of arachidonic acid (AA).

Total patient no: n=1002	**AA Q1** n=253		**AA Q2** n=248		**AA Q3** n=255		**AA Q4** n=246		**P-value for trend**
MACE	61	(24.1%)	41	(16.5%)	48	(18.8%)	58	(23.6%)	0.97
AMI	17	(6.7 %)	15	(6.0 %)	18	(7.1 %)	28	(11.4 %)	0.05
Revasc	21	(8.3%)	16	(6.5%)	16	(6.3%)	24	(9.8%)	0.59
Stroke	11	(4.3 %)	5	(2.0 %)	10	(3.9 %)	5	(2.0 %)	0.35
HF	13	(5.1 %)	13	(5.2 %)	10	(3.9 %)	9	(3.7 %)	0.33
Death	16	(6.3 %)	13	(5.2 %)	9	(3.5 %)	17	(6.9 %)	0.99

AA= Arachidonic Acid. MACE = Major Adverse Clinical Events (nonfatal MI, unscheduled coronary revascularization, stroke, all-cause death, or hospitalization for new or worsened heart failure). AMI = Acute Myocardial Infarction. Revasc = Revascularization (percutaneous coronary intervention or coronary artery bypass grafting). HF = Heart Failure. P-values for trends across the quartiles.
